# The Role of GABA and Its Receptors in Temporal Lobe Epilepsy

**DOI:** 10.3390/biom16030422

**Published:** 2026-03-12

**Authors:** Günther Sperk, Susanne Pirker

**Affiliations:** 1 Department of Pharmacology, Medical University Innsbruck, 6020 Innsbruck, Austria; 2 Department of Neurology, Klinik Hietzing, 1130 Vienna, Austria; 3 Karl Landsteiner Institute for Clinical Epilepsy Research and Cognitive Neurology, 1130 Vienna, Austria

**Keywords:** GABA, mesial temporal lobe epilepsy, GABA_A_ receptor, GABA_B_ receptor, hippocampus, CA1, CA2, mossy fiber, kainic acid, pilocarpine, kainic acid receptor, GluK1, GluK2

## Abstract

Mesial temporal lobe epilepsy (TLE) is the most common and severe form of focal epilepsy. This review examines the diverse mechanisms by which the GABAergic system contributes both to seizure generation and to protective processes that limit epileptogenesis and seizure progression in TLE. We focus on findings from established animal models of TLE as well as studies of surgically resected tissue from patients who had undergone therapeutic intervention. Experimental models include sustained electrical stimulation of the perforant path, as well as the kainic acid (KA) and Li-pilocarpine models. Although these paradigms induce status epilepticus (SE) through distinct mechanisms, they ultimately converge on prolonged excitation of hippocampal CA3 pyramidal neurons and interconnected regions of the hippocampus and broader limbic network. In response to epileptic seizures, GABA synthesis is enhanced, as evidenced by the marked upregulation of the GABA-synthesizing enzymes GAD65 and GAD67, along with their ectopic expression in glutamatergic mossy fibers of the hippocampus. Shortly after acute seizures, a transient expression of the embryonic GAD67 splice variant, GAD25, is observed, although its functional significance remains unclear. At the receptor level, animal models of TLE show upregulation of GABA_A_ receptor subunits α2, α4, β3, and γ2, accompanied by downregulation of α5 and δ subunits, suggesting reduced tonic inhibition. In contrast, hippocampal tissue from patients with TLE exhibits pronounced upregulation of α5 and δ subunits, indicative of enhanced extrasynaptic tonic inhibition. Similarly, whereas GABA_A_ receptor subunits are mildly downregulated in animal models, they are consistently upregulated across hippocampal subfields in human TLE, pointing toward strengthened GABAergic inhibition. Conversely, genetic variants of GABA_A_ receptor subunits and autoantibodies targeting these receptors can contribute to the etiology of epilepsy, often with onset in childhood. Moreover, degeneration or functional silencing of specific GABAergic interneuron populations—such as parvalbumin-positive neurons in the subiculum—can induce epilepsy in rodent models and is likewise associated with TLE in humans.

## 1. Introduction

Epilepsies represent a complex group of neurological disorders characterized by varied causes, clinical features, and outcomes. According to the International League against Epilepsy (ILAE), the clinical landscape of epilepsies include a wide array of conditions that differ not only in their etiology but also in their manifestations and outcome [1]. In Europe, approximately between 2.6 and 6 million individuals are affected by epilepsy, corresponding to a lifetime prevalence of about 3.3–7.8 per 1000 persons [2]. Among focal epilepsies, mesial temporal lobe epilepsy (TLE) represents the most prevalent subtype, accounting for approximately 60–80% of cases [3,4].

The clinical presentation of epilepsy is highly syndrome-specific. Seizure onset frequently occurs during infancy or early childhood and, in certain syndromes, may remit over time. In contrast, seizures associated with TLE are typically chronic and persist throughout adulthood [5]. Seizure duration varies considerably: some episodes last only a few seconds, whereas others may extend for several minutes. On average, generalized tonic–clonic seizures persist for approximately 57–102 s [6]. Seizures lasting longer than five minutes are defined as status epilepticus (SE), a neurological emergency requiring immediate medical intervention to prevent irreversible neuronal injury.

## 2. Temporal Lobe Epilepsy (TLE)

TLE is among the most common and severe epilepsy syndromes. It is frequently preceded by precipitating insults such as prolonged febrile seizures, traumatic brain injury, cerebral ischemia, or SE. Notably, following initial febrile seizures, a latent period of several years may elapse before the clinical manifestation of TLE [7,8]. In patients older than 40 years, limbic encephalitis has been increasingly recognized as a potential etiological factor [3,9].

Clinically, TLE is often refractory to pharmacological treatment, and a substantial proportion of patients develop drug-resistant epilepsy [10,11]. Typically, there is marked degeneration of CA1 and CA3 pyramidal neurons and interneurons within the dentate hilus, whereas dentate granule cells, CA2 pyramidal neurons, and the subiculum are relatively preserved [12,13,14]. In most cases, these pathological alterations are unilateral. Despite this focal pathology, many patients remain resistant to conventional antiepileptic drug therapy. For individuals with medically intractable TLE, surgical resection of the epileptogenic focus represents a potentially effective therapeutic option (e.g., amygdalohippocampectomy) [15]. Tissue originating from surgical resection is a valuable object for research on TLE.

The molecular mechanisms governing the generation and termination of seizures remain poorly understood. Among the key processes implicated in seizure pathophysiology is dysfunction of the γ-aminobutyric acid (GABA) ergic system. GABA serves as the principal inhibitory neurotransmitter in the central nervous system and plays a critical role in maintaining the balance between neuronal excitation and inhibition. It is primarily released by inhibitory interneurons and exerts its effects via ionotropic GABA_A_ and metabotropic, GABA_B_ receptors, thereby suppressing excitatory neuronal activity. Dysregulation of GABAergic signaling can disrupt this excitatory–inhibitory balance, resulting in hyperexcitability and seizure generation [16,17,18,19].

This review aims to examine the role of the GABAergic system in both the initiation and termination of seizures, with particular emphasis on the mechanisms by which GABA signaling contributes to seizure cessation. Since TLE is the most frequent and most severe form of epilepsy, we focus this review on epilepsy-induced changes in animal models of TLE and human TLE.

## 3. Animal Models of Temporal Lobe Epilepsy (TLE)

There are two main groups of animal models related to TLE (For review see [20]):

1. Kindling models and 2. Models in which recurrent seizures follow a SE.

### 3.1. Kindling Models

Kindling is mostly performed in rats or mice. The animals are typically stimulated by a subthreshold electrical or chemical stimulation (e.g., pentylenetetrazol) daily for about three weeks. The electrical stimulation is typically performed in the hippocampus, amygdala or piriform cortex. After-discharges are characterized as prolonged, high amplitude rhythmic discharges in the electroencephalogram (EEG). Repeated sub-threshold stimuli cause an increase in after-discharge duration, accompanied by evolving behavioral seizures with increasing severity and duration. Rats are considered as “fully kindled” when they respond with generalized seizure to the stimulus [21]. The animals, however, in contrast to the SE models, usually do not develop spontaneous seizures. For rapid kindling rats are stimulated several times per day.

### 3.2. TLE Models Based on Induction of a Status Epilepticus (SE)

#### 3.2.1. Models Using Sustained Electrical Stimulation

*Electrical Stimulation of the Perforant Path*. Sloviter et al. used sustained electrical stimulation of the perforant path for inducing an SE that was neuropathologically associated with the typical signs of TLE, including damage of dentate pyramidal basket cells, hilar cells in general and CA3 and CA1 pyramidal cells. CA2 pyramidal cells and dentate granule cells were relatively unaffected [22,23,24].

*Electrical Stimulation of the Hippocampus*. Lothman and his group used sustained unilateral electrical stimulation of the hippocampus for producing self-sustaining limbic SE [25]. The SE was associated with degeneration in CA1 [26].

#### 3.2.2. TLE Animal Models Using Neurotoxic Treatment

##### The Kainic Acid (KA) Model

*Application of KA.* The concept of *excitotoxicity*—the notion that excessive exposure to excitatory amino acids induces neuronal damage—was first introduced by John W. Olney [27,28]. He subsequently applied kainic acid (KA), a potent excitatory toxin isolated from the seaweed *Digenea simplex*, which acts primarily through KA receptors and induces a *SE*, ultimately leading to severe neurodegeneration. For review see [29].

KA-induced lesions prominently affect CA1 and CA3 pyramidal neurons of the hippocampus, closely resembling Ammon’s horn sclerosis observed in TLE. Mossy cells (giving rise to associational–commissural fibers) are also vulnerable, whereas CA2 pyramidal neurons, dentate granule cells, and most parts of the subiculum are relatively spared [30]. Additional damage occurs in thalamic nuclei [31] and in layer III of the medial entorhinal cortex, which projects to CA1 and the subiculum via the temporoammonic pathway [32].

KA has been administered using various routes. Schwob and colleagues applied KA intraperitoneally (8–12 mg/kg). However, focal administration has also been employed, including intra-amygdaloid injection (1 µg) [33,34,35], intraventricular injection [36], intrahippocampal injection [37], and subcutaneous administration [38].

KA only poorly penetrates the blood–brain barrier (BBB), as evidenced by the substantial difference between systemic and intracerebral doses required to elicit comparable effects. Using tritiated KA, Berger and colleagues demonstrated that only 2.6–8.2% of the toxin enters the brain following systemic administration [39]. This limited penetration complicates dose–response analyses and pharmacological investigations [40]. Furthermore, seizure activity itself may transiently disrupt the BBB [41], further contributing to variability.

*Status epilepticus induced by KA.* The acute SE-induced by KA typically lasts 12–24 h. Following this period, animals recover physically and regain body weight. However, they subsequently develop recurrent spontaneous seizures over the following weeks and are generally considered chronically epileptic after 2–4 weeks [42,43].

Mechanistically, KA strongly activates the excitatory mossy fiber pathway projecting to CA3 pyramidal neurons. Excitation is initiated primarily via postsynaptic GluK1/GluK2 receptors in the CA3 region [44,45]. Synaptic silencing of the CA3 synapse attenuates seizure propagation [46]. From CA3, epileptiform activity propagates to CA1 and subsequently to other limbic structures like the subiculum and particularly to the entorhinal cortex, which provides feedback projections to the dentate gyrus. Thus, the CA3 region acts as a pacemaker for synchronized network activity [44].

Neuronal damage may additionally involve anoxic–ischemic mechanisms [38], likely mediated by the high density of NMDA, AMPA, and KA receptors throughout the hippocampus [47,48].

*Propagation of Acute Seizures by KA.* In the hippocampus, two principal KA receptor subunits predominate: GluK1 (formerly GluR5) and GluK2 (formerly GluR6). GluK1-containing receptors are primarily expressed on interneurons, whereas GluK2-containing receptors are preferentially located on principal cells. These receptor subunits undergo RNA Q/R editing, which critically influences their functional properties [49,50,51].

Unedited GluK2(Q) receptors function as high-conductance ion channels mediating fast excitatory transmission. In contrast, edited GluK2(R) receptors exhibit metabotropic properties. Their activation triggers a signaling cascade involving pertussis toxin–sensitive G_i/o_ proteins, phospholipase C, and protein kinase C, ultimately inhibiting GABA release from interneurons [52,53,54].

GluK1 receptors are also expressed presynaptically on terminals of GABAergic interneurons in CA1. These receptors are located both in axonal compartments and in somato-dendritic regions [54,55]. Activation by glutamate—but not by AMPA or NMDA receptors—increases quantal GABA release from CA1 interneurons [56]. At interneuron–interneuron synapses, KA facilitates tonic GABA release and thereby enhances the inhibitory drive [57], partially counterbalancing its excitatory effects. However, also repetitive action potential firing of interneurons stimulated by glutamate or KA through GluK1 receptors results in drastically increased tonic inhibition of CA1 pyramidal neurons at their somata and apical dendrites, protecting them from over-excitation [57].

At these interneuron–principal cell synapses, KA exerts biphasic effects on its presynaptic receptors; for review see [58]). High concentrations (approximately 50 µM) suppress GABA release (through GluK1(R) receptor subtype) [59,60], whereas low concentrations (approximately 0.5 µM) or the GluK1-selective agonist ATPA enhance GABA release (through the ionotropic subtype of the KA receptor; GluK1(Q) subtype) [61,62]; for review see [63,64].

*Developing spontaneous recurrent seizures.* Starting about one week after the acute *SE*, the rats present spontaneous recurrent seizures, thus become epileptic [42,43]. One possible mechanism may be the sprouting of mossy fibers.

Mossy fibers originating from dentate granule cells play a central role in the initiation and propagation of KA-induced excitation. High-affinity KA receptors are densely expressed presynaptically on mossy fiber terminals [65,66], rendering them highly susceptible to modulation of glutamate release. Following degeneration of hilar mossy cells—which normally project to the inner molecular layer—mossy fibers undergo aberrant sprouting into the inner molecular layer of the dentate gyrus [66,67,68,69]. These sprouted fibers extensively innervate granule cell dendrites, effectively replacing mossy cell terminals. A minority of aberrant mossy fiber synapses also form on interneuron dendrites [67]. Sprouted mossy fibers may have a role in the development of recurrent seizures.

Mossy fibers sprout also in human TLE patients, provided Ammon’s horn sclerosis has developed. There are, however, a certain percentage of patients with TLE, undergoing amygdalahippocampectomy but do not present sprouted mossy fibers [12].

##### The Lithium–Pilocarpine Model

John W. Olney also made seminal contributions to the discovery of the convulsive properties of cholinomimetic agents [70]. Subsequently, Waldemar A. Turski and colleagues established the pilocarpine model of TLE, demonstrating its characteristic behavioral, electroencephalographic, and neuropathological features [71]. Systemic administration of pilocarpine induces seizures and *SE* in rats and mice, followed by widespread neuronal damage resembling that observed in the KA model. Affected regions include the hippocampus, amygdala, thalamus, and substantia nigra. In contrast to the KA model, seizure generation in the pilocarpine model is strongly influenced by basal ganglia structures, including the striatum–putamen, substantia nigra, endopeduncular nucleus, and nucleus accumbens [71,72]. Subsequently, regions particularly vulnerable to anoxia/ischemia—such as the hippocampus, amygdala, and thalamus—become prominently involved.

The later introduction of lithium pretreatment significantly lowered the threshold for pilocarpine-induced seizures and exacerbated seizure-associated brain damage [73,74]. To minimize peripheral cholinergic side effects, methyl-scopolamine is routinely co-administered.

The lithium–pilocarpine model in rats and mice is now as widely used as the KA model and remains one of the most established experimental paradigms for studying TLE pathophysiology and epileptogenesis.

## 4. The GABAergic Synapse

### 4.1. Synthesis of GABA

GABA is produced in the nervous system from glutamate by the enzyme glutamate decarboxylase (GAD) with pyridoxal phosphate as a cofactor. There are two isoforms of GAD with different molecular weights and cofactor binding properties: GAD65 (65 kDa) [75,76,77] binds pyridoxal phosphate loosely. Its activity is regulated by the availability of the cofactor pyridoxal phosphate and through gene expression (de novo synthesis). GAD67 (67 kDa) has pyridoxal phosphate tightly bound and is normally active as a holoenzyme.

### 4.2. Storage and Release of GABA

After synthesis, GABA is transported into synaptic vesicles by the vesicular GABA transporter (VGAT) [78]. VGAT is specific for GABA and does not import other amino acids like glutamate except glycine. During an action potential, calcium ions enter the presynaptic neuron, triggering the fusion of vesicles with the membrane and the release of GABA into the synaptic cleft. GABA is also frequently stored in the same neurons together with neuropeptides in large dense core vesicles. For their release, these require higher frequency stimulation than synaptic vesicles. Neuropeptides have their own receptors and have important roles in modulating transmission of GABA interneurons [79]. Together with different calcium binding proteins neuropeptides are often used for classification of subtypes of GABAergic interneurons [80].

### 4.3. GABA Receptors

GABA exerts its inhibitory effects through two main receptor types: (1) GABA_A_ receptors are ionotropic chloride channels primarily located on the postsynaptic membrane. Binding of GABA opens to Cl^−^ ions. When the extracellular chloride concentration is higher, Cl^−^ flows into the postsynaptic neuron, causing hyperpolarization [81]. This chloride gradient is mainly maintained by the potassium-chloride co-transporter KCC2 [82].

(2) GABA_B_ receptors are metabotropic G-protein-coupled receptors found both post- and pre-synaptically. Their activation leads to a slow, prolonged inhibition: they open G-protein-activated inwardly rectifying potassium (GIRK) channels, enhancing hyperpolarization, and inhibit voltage-gated calcium channels, reducing excitability [83,84].

### 4.4. Termination of the Action of GABA

GABA’s effects are terminated by its rapid removal from the synaptic cleft via specific transporters. The two main GABA re-uptake transporters are GAT-1 and GAT-3 [85]. They return GABA to presynaptic neurons and astrocytes, respectively. In neurons, the reabsorbed GABA is directly recycled into vesicles. In astrocytes, GABA is degraded by GABA transaminase and fed into metabolic pathways (e.g., the citric acid cycle) or is converted into glutamine and glutamate, which are recycled to the neuron and used as substrate for GABA synthesis.

## 5. Impaired GABA Transmission as a Cause for Epilepsy

### 5.1. Antagonists and Agonists of the GABA_A_ Receptor

GABA_A_ receptor antagonists such as bicuculline—a competitive inhibitor at GABA_A_ receptors—and picrotoxin, which binds within the ion channel pore, exert potent proconvulsant effects. Also, antibodies to the GABA synthesizing enzyme GAD can be associated with TLE [86]. In contrast, agonists of the GABA_A_ receptor, including benzodiazepines, barbiturates, and the GABA transaminase inhibitor vigabatrin, display robust anticonvulsant properties. In patients, baclofen a GABA_B_ receptor agonist exerts a spasmolytic action but can also provoke convulsions [87,88].

### 5.2. Disrupted GABAergic Transmission as a Mechanism Underlying TLE

Impairments in GABAergic neurotransmission have been proposed as a key pathomechanism in TLE, as demonstrated across multiple experimental models. According to Sloviter’s “dormant basket cell” hypothesis [89], the selective loss of excitatory mossy cells in the dentate gyrus reduces their activation of inhibitory GABAergic basket cells. This disinhibition facilitates seizure generation, as observed in TLE models involving chronic electrical stimulation. Direct alterations of the functions of certain subpopulations of interneurons during epileptogenesis have also been proposed. Thus, Andrioli et al. (2007) reported selective reduction in parvalbumin-positive interneurons in the subiculum of TLE patients, even in the absence of hippocampal sclerosis [90]. A finding that was recapitulated in two rodent models of TLE-induced by pilocarpine or KA, demonstrating a targeted loss of parvalbumin-positive neurons in the outer molecular layer of the subiculum [31,91]. On the other hand, innervation of CA1 pyramidal cells by parvalbumin-positive neurons remains unchanged in human TLE tissue, indicating sprouting of these neurons [92].

### 5.3. Impairing Specific Subpopulations of GABAergic Neurons Induces TLE

**Figure 1:**
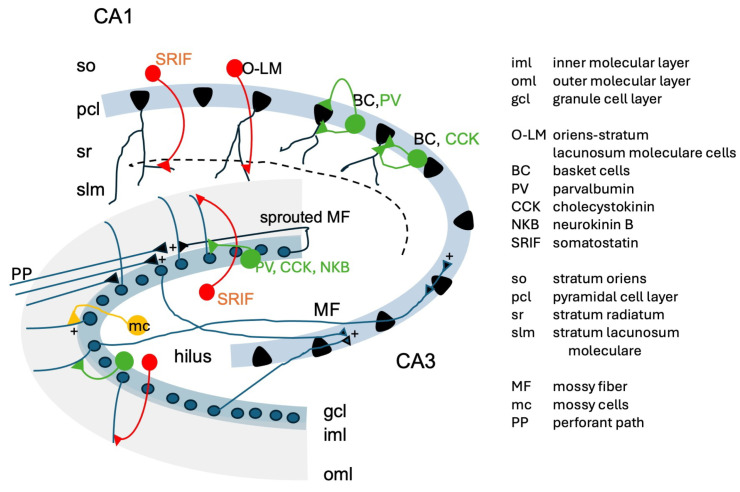
Simplified scheme of the hippocampus: The main excitatory neurons (black) are the granule cells (gcl) that are innervated in the inner molecular layer by perforant path neurons (PP), coming from the entorhinal cortex. Granule cells project through their mossy fibers (MF) to CA3 pyramidal neurons, which project via the Schaffer collaterals to CA1 (not included). Within the hilus there are also the excitatory mossy cells (mc; orange) that project via the associational commissural fibers to the inner molecular layer. They are highly vulnerable in TLE. There are several classes of interneurons in the dentate gyrus: basket cells (BC) containing, in addition to GABA, parvalbumin, CCK-8 or NKB (green). They innervate granule cells at their perikarya. Somatostatin neurons (red) project to the outer molecular layer and contain also NPY. In the hippocampus proper (Ammon’s horn) there are two major classes of interneurons, basket cells (containing parvalbumin, PV) or CCK-8 (green); they form axo-axonic synapses and mediate feed-forward inhibition, and somatostatin containing O-LM cells (projecting from the stratum **o**riens to the stratum **l**acunosum **m**oleculare); they mediate feedback inhibition upon pyramidal neurons. Both classes of neurons are crucial in the generation of limbic seizures [32,33,34]. Not included are PV containing axo-axonic neurons, interneuron-specific interneurons, containing VIP and calretinin and NPY-containing Yvi cells; see for details [80].

Evidence that a specific loss of GABAergic interneurons can be causative for induction of TLE has been provided by Drexel et al. [31,93,94,95,96]. Using viral vectors selectively blocking GABA release from parvalbumin-positive interneurons in the subiculum and CA1 region without inducing cell death they observed spontaneous tonic–clonic seizures, mimicking key features of TLE [94]. Similarly, selective silencing of somatostatin-positive interneurons, which mediate feedback inhibition, led to recurrent seizures [42,43] highlighting their critical physiological role in seizure suppression [93] (see Figure 1). On the other hand, silencing vasoactive intestinal peptide (VIP)-containing neurons in the subiculum using the same approach produced anti-convulsive effects [96].

## 6. The GABA System in Response to Seizures and Epilepsy

### 6.1. The GABA Synthetizing Enzymes Glutamate Decarboxylase (GAD) in Epilepsy

To investigate the role of GABAergic neurons in chronic epilepsy, we examined levels of GAD as well as somatostatin and neuropeptide Y (NPY), both of which are co-expressed with GABA in specific interneuron populations. Subcutaneous injection of KA in rats induced SE and triggered symptoms resembling TLE, characterized by spontaneous, recurrent seizures and a reduced seizure threshold to the convulsant pentylenetetrazol [97,98].

GAD activity was assessed in the frontal cortex—an area not affected by neurodegeneration—and thirty days after KA injection a significant 26% increase in Vmax (referring to the number of enzyme particles) was observed, with no change in the enzyme’s affinity for its substrate glutamate at saturating concentrations (16 mM) of the cofactor pyridoxal phosphate [98]. At the same time, somatostatin and NPY levels increased significantly (by 60% and 135%, respectively) in the frontal cortex. These increases were dependent on the initial seizure severity and were similarly observed in rats kindled with pentylenetetrazol for 28 days [99]. Because somatostatin and NPY are expressed in GABAergic interneurons [100], their upregulation reflects enhanced interneuron activity during kindling and in epilepsy. Upregulation and sprouting of GAD, NPY and somatostatin has also been shown in the hippocampus of TLE patients [8,101].

These findings were later substantiated at the mRNA level: first by demonstrating increased GAD65 mRNA expression [102], and subsequently—after cloning of GAD67 [103]—by showing elevated GAD67 mRNA expression in the hippocampus of rats treated with KA or Li-pilocarpine, and in spontaneous epileptic rats [104,105,106].

This pronounced overexpression of GAD, and the resulting increased GABA synthesis, likely represents a powerful endogenous anticonvulsant mechanism, possibly accounting for the reduction in seizure frequency from almost continuous seizures during the *SE* to 2 to 5 convulsions per week. Notably, GAD activity continued to rise over the thirty days after KA administration [98], suggesting that the spontaneous seizures occurring after *SE* further stimulated enzyme synthesis.

### 6.2. Sprouted Mossy Fibers Innervate Basket Cells and/or Granule Cells

Granule cells and their mossy fibers comprise a major input to the hippocampus. They contain glutamate as principal transmitter and expose high morphological and biochemical plasticity. A second class of glutamatergic neurons, located in the dentate hilus are mossy cells [107,108]. They give rise to associational and commissural fibers that project to the inner molecular layer of the ipsi- and contralateral dentate gyri. Mossy cells excite basket cells but are highly vulnerable and degenerate rapidly in TLE and corresponding animal models. Their loss prompts sprouting of granule cell axons (mossy fibers) into the terminal zones formerly occupied by mossy cells (inner molecular layer), leading to the formation of aberrant synapses with granule cell dendrites [36] and/or interneurons [67,109]. These changes could contribute to network hyperexcitability or, paradoxically, to an augmented inhibitory tone, depending on the neuron innervated by sprouted mossy fibers (see Figure 1).

### 6.3. GAD and GABA in Excitatory Granule Cells and Mossy Fibers

Sandler and Smith demonstrated that mossy fibers—in addition to glutamate—contain also GABA and GAD in macaque monkeys [110]. In animal models of TLE [105,111,112] and in human TLE [106], GAD is markedly upregulated in granule cells/mossy fibers. Several hypotheses have been formulated to interpret the physiological consequences:

- Co-release of GABA and glutamate during seizures [113]
- Reversal of GABA transport due to seizure-induced ionic shifts [114]
- Buffering of excess of glutamate accumulated in granule cells during seizures by its conversion to GABA

We favor the third hypothesis. Granule cells, even under epileptic conditions, do not express the vesicular GABA transporter (VGAT) [112], making synaptic GABA release unlikely (contradicting hypothesis 1). While seizure-related sodium influx might drive reversed transport via glutamate and GABA transporters [115], expression of GAT-1 and GAT-3 is generally reduced following KA treatment—except for a transient upregulation at 9 h—casting doubt on hypothesis 2. Instead, intracellular conversion of glutamate to GABA could serve as an effective mechanism to buffer excessive glutamate during seizures by its conversion to GABA. GABA, however, would remain in the cytoplasm, since it is not transported into vesicles due to the lack of the VGAT in granule cells. Anyway, all these mechanisms would imply augmented GABAergic function. Furthermore, GABA transaminase mRNA expression becomes reduced at the same time [112]. The enzyme is responsible for GABA degradation in astrocytes, indicating impaired degradation of GABA in the hippocampus sustaining the increase in intracellular GABA.

### 6.4. Embryonic GAD67 Splice Variant in Granule Cells

Granule cells express an embryonic splice variant of GAD67 following KA-induced seizures [105]. This variant lacks a portion of exon 7, resulting in an enzymatically inactive protein (GAD25). When we observed GAD expression in granule cells, we wondered whether it corresponded to the previously described embryonic splice variant. However, our findings showed that both, the embryonic and adult forms of GAD67 are expressed in the KA model [105]. Notably, the embryonic variant peaks earlier than the adult form (2 vs. 6 h post-KA; [116]), mirroring gene expression patterns observed during development.

The physiological role of GAD25 remains unclear. However, it is worth noting that GAD67 knockout mice develop a cleft palate and die shortly after birth [117], while GAD65 knockout mice exhibit only a mild increase in seizure susceptibility. It is likely that GAD25 is also not expressed in GAD67 knockout mice, leading to speculation that GAD25 might play a role in the regulation of embryonic development, and its loss could contribute to the development of cleft palate.

## 7. GABA_A_ Receptors in TLE Animal Models

### 7.1. Assembly of GABA_A_ Receptor Subunits

GABA_A_ receptors are the principal inhibitory receptors in the brain. They are composed of five subunits that form a chloride ion channel [118,119]. The subunits are derived from multiple gene families (α1–α6, β1–β3, γ1–γ3, δ, ε, θ), allowing for the formation of various GABA_A_ receptor types in the mammalian brain [120,121,122]. Despite the wide range of theoretically possible GABA_A_ receptor configurations, the number of actual receptor pentamers is limited. In mammals, the basic structure of GABA_A_ receptors typically consists of two α-subunits, two β-subunits, and either one γ- or δ-subunit [123]. The GABA binding site is located at the interface between the α- and β-subunits, while the benzodiazepine binding site, which enhances GABA’s affinity for the receptor, is situated at the interface between an α- and γ-subunit. Notably, GABA_A_ receptors containing α4 or δ-subunits do not respond to benzodiazepines. When the receptor is stimulated, the channel opens, allowing chloride ions (Cl^−^) to enter the neuron, causing rapid hyperpolarization. This chloride gradient is maintained by the chloride exporter KCC2 and counteracted by the chloride importer NKCC1 (discussed below).

There exist numerous different variants of GABA_A_ receptor subunits that have been associated with epilepsy. They can cause complex changes to receptor properties resulting in various degrees of gain-of-function (See the excellent review by Absolon et al. [124]). For example, variants in the α1, α4, β3, γ2, or δ subunits, or a deficiency in the β3 subunit, are associated with different forms of generalized epilepsies [125,126,127,128,129].

### 7.2. GABA_A_ Receptor Binding Studies in Kindling and TLE Models

In vitro GABA_A_ receptor binding studies use a variety of ligands, including [^3^H]-GABA, [^3^H]-muscimol, and [^3^H]-labeled benzodiazepines. Muscimol binds selectively to a subgroup of GABA_A_ receptors that contain the δ-subunit, which are predominantly expressed at extrasynaptic sites [130]. [^3^H]-labeled benzodiazepines, such as [^3^H]-diazepam or [^3^H]-flunitrazepam, are commonly used in research, but they specifically label γ2-containing receptors, leaving receptors containing α4, α6, or δ subunits unlabeled. In human studies, positron emission tomography (PET) typically uses [^11^C]-flumazenil or [^18^F]-flumazenil as ligands to assess GABA_A_ receptor binding [130,131,132].

Neurochemical and electrophysiological studies in animal models suggest that GABA_A_ receptor function is fundamentally altered in the hippocampus in TLE [133,134,135,136]. Reduced binding of GABA_A_ receptor ligands, such as [^3^H]-muscimol or [^3^H]-benzodiazepines, has been observed in the hippocampus following SE-induced by lithium/pilocarpine or KA. These reductions correlate with significant hippocampal damage, although granule cells, CA2 pyramidal cells, and the subiculum remain largely preserved [9].

In contrast, in the kindling model, there are increases in GABA_A_ receptor density in the molecular layer of the dentate gyrus, which contains granule cell dendrites [137,138]. This layer is rich in GABA_A_ receptors and plays a critical role in regulating excitatory input to the hippocampus. The receptors in this region receive input from hilar interneurons, including: (1) parvalbumin, cholecystokinin-octapeptide (CCK-8), and neurokinin B (NKB)-containing basket cells, which form synapses on granule cells and in the inner molecular layer [80], and (2) somatostatin- and NPY-containing interneurons, which innervate the outer molecular layer. Both cell types play essential inhibitory and regulatory roles [80,107].

In the CA1 region, GABA_A_ receptor binding is reduced after kindling, despite the absence of neuronal loss [138,139].

### 7.3. Changes in GABA_A_ Receptor Subunit mRNAs During Kindling

*Kindling.* As described in Section 3.1, kindling is a process in which repeated sub-threshold electrical or chemical stimulation progressively lowers the seizure threshold in rodents, typically requiring 10–20 sessions. Although kindling reduces the seizure threshold, it does not lead to spontaneous seizures, making it a model for studying seizure susceptibility rather than epilepsy. Neurochemical changes observed after a single stimulation reflect only the acute response to the stimulation, whereas changes induced by repeated stimulations more closely resemble those seen in animal models of TLE. One of the key advantages of the kindling model over TLE models is the absence of neurodegeneration. Early studies examined the effect of kindling on GABA_A_ receptor subunit expression. Kamphuis et al. assessed the expression of 10 GABA_A_ subunit mRNAs in the granule cell layer and CA1/CA3 pyramidal neurons of rats kindled for 6, 14, or 30 days at the Schaffer collateral/commissural fibers [140,141]. After 6 and 14 days of kindling, there were significant increases in α1, α2, α3, α4, β1, β2, β3, and γ2 mRNAs (14–36%), while δ mRNA decreased insignificantly; α5 mRNA showed a slight reduction. Notably, in fully kindled rats (after 28–30 days), most changes subsided, except for persistent increases in β2, β3, and γ2 mRNAs (Table 1). Practically identical results were observed by Kokaia et al. for subunits β3, and γ2 and by Nishimura et al. showing increases in α4, γ2, β2 and β3 and δ in granule cells of the dorsal and ventral hippocampus after two and eight electrical kindling sessions [142,143]. In CA1 pyramidal neurons, α2, β3 and γ2 were consistently upregulated, [140,141,143]. These changes largely disappeared after a 28-day seizure-free period, except for sustained increases in α3 and γ2 mRNA levels and the decreases in δ-subunit in granule cells and an increase in γ2 in CA3 pyramidal neurons.

A landmark study by Nusser et al. used immunoelectron microscopy to explore synaptic mechanisms [155]. They observed increased densities of α1, α2, β2/β3, and γ2-containing receptors at perisomatic synapses in the dentate molecular layer of kindled rats. These changes were accompanied by an enlarged synaptic junctional area and a 66% increase in quantal synaptic currents, suggesting enhanced GABAergic transmission and improved synchronization of GABA_A_-mediated signals.

### 7.4. Changes in GABA_A_ Receptor mRNAs and Immunoreactivity (IR) in the Kainic Acid and Li-Pilocarpine TLE Models

*Animal TLE models.* The most commonly used animal models for TLE include the pilocarpine model [156], often combined with lithium [73] and methyl scopolamine to reduce peripheral side effects, the KA model in rats and mice [157], and sustained electrical stimulation of the hippocampus or amygdala in rats [158]. In all of these models, *SE* leads to neuronal loss followed by the development of spontaneous seizures, which is characteristic of epilepsy. This neuronal loss resembles hippocampal sclerosis seen in humans and shares features of ischemic damage [159]. It includes hippocampal sclerosis, characterized by CA1 and CA3 pyramidal cell loss and hilar interneuron degeneration whereas granule cells, CA2, and the subiculum are relatively spared [157,158,160]. Although administration of diazepam during *SE* can reduce neuronal death, it does not prevent the subsequent development of spontaneous seizures and epilepsy [97]. The earliest time points (3–12 h after KA administration) likely reflect acute responses to *SE*, including ischemic injury and neurodegeneration [38]. After approximately 48 h, acute seizures subside, and recurrent spontaneous seizures (occurring 2–5 times per week) develop. By day 30, rats typically have entered a chronic epileptic state, which corresponds to the TLE seen in human patients. Subunit changes in GABA_A_ receptors may vary across these different stages.

#### 7.4.1. Changes in GABA_A_ Receptor Assembly in Dentate Granule Cells

GABA_A_ receptor expression is highly heterogeneous in control rats. Subunits α1 and γ2 are broadly expressed across the hippocampus [161], with α5 being the most abundant α-subunit in the Ammon’s horn, but less so in the dentate gyrus. Subunits β1 and β3 are expressed throughout CA1 and CA3. In granule cells, subunits α2, α4, β3, and to a lesser extent β1 are enriched, alongside δ, which is more abundant in granule cells than in pyramidal cells [161]. Consequently, predominant receptor assemblies include α5β3γ2 and α1/α2β3γ2 in pyramidal dendrites, and α2β3/β1γ2 and α4β3δ in the dentate molecular layer. Receptors containing α5β3γ2 and α4β3δ are extra-synaptic and mediate tonic inhibition, whereas α1/α2β3γ2 receptors are synaptic and mediate phasic inhibition [162].

**Figure 2:**
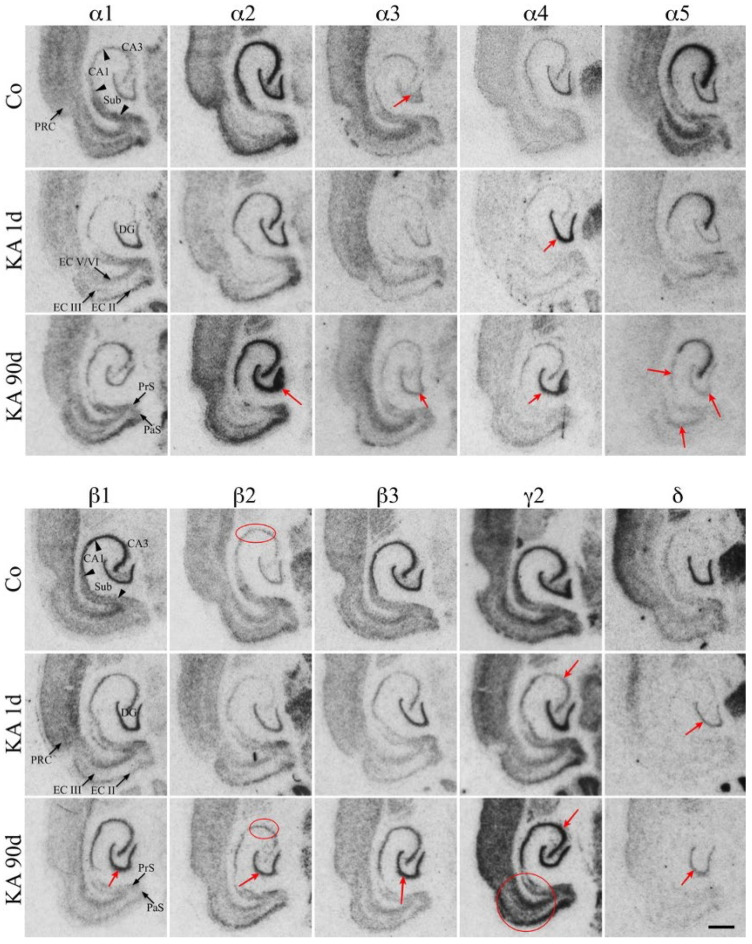
In situ hybridization of 13 GABA_A_ receptor subunits in the ventral hippocampus of male rats treated with KA. Controls, 1 day and 30 days after KA injection. Red arrows demark pronounced changes in individual areas, as also mentioned in the text. α2: marked increase in granule cells 90 days after KA; α3, interneurons in the hilus of controls; α4, increased expression in granule cells after KA; α5, spectacular losses after KA; β2 increased expression in CA1 interneurons and granule cells; β1, β3, increased expression in granule cells; γ2, transient reduction in CA3 pyramidal cells. The figure was originally published by Drexel et al., Front Neural Circuits (2013) [145]. It is openly licensed via CC BY 4.0. Red circles mark changes of β2 in sector CA1 and of γ2 in the entorhinal cortex. Abbreviations: DG, dentate gyrus; Sub, subiculum; PRC, perirhinal cortex; Sub, subiculum; PrS, presubiculum; PaS, parasubuculum; EC, entorhinal cortex layers II, III, IV and V; CA1, CA3, cornu Ammonis 1 and 3. Scale bar: 0.5 cm.

*α-subunits.* In granule cells, α2, α3, and α5 mRNAs decreased 6–24 h after KA, while α1 and α4 increased transiently during and shortly after *SE* [144]. Only α2 showed a clear reduction in IR after 12 h [146]. At chronic stages (7–30 days), mRNA for α1, α3, and α4 were slightly increased, α2 mRNA levels returned to baseline (α2-IR was increased after 90 days), and α5 mRNA and IR (Table 1 and Figure 2) remained significantly reduced. Similar findings were reported for Li-pilocarpine, except that α1 decreased [147]. On the protein level, all α-subunit IR were enhanced in the molecular layer of the dentate gyrus (notably α1 and α2), but not α5 [146,150].

*β-subunits.* Subunit β3 was the most prominently expressed β-subunit. All β mRNA levels initially decreased 6 to 24 h after KA injection, followed by marked increases in β1, β2 and β3 mRNAs and IR levels after 7–90 days (Table 1, Figure 2) [144,147].

*γ2- and δ-subunits*. Subunit γ2 mRNA decreased by ~60% during the acute *SE* (6 h after KA) but recovered thereafter. γ2-IR then increased at 24 h and 30 days in the molecular layer. In contrast, δ mRNA and IR decreased at all time intervals [143,144,145,146,148].

#### 7.4.2. Changes in GABA_A_ Receptor Subunits in Pyramidal Cells

Interpretation of data on changes in α4 in pyramidal cells is complicated by the extensive neuronal loss. At early intervals, α5, β3, and γ2 mRNAs consistently decreased in CA1 and CA3 [144,145]. Protein analyses confirmed reduced β3-IR, whereas γ2 showed only a marginal increase [146]. At chronic stages (30 days after KA), mRNA levels of α1, α2, α4, all β-subunits, and γ2 were reduced, likely reflecting the extensive cell loss. Subunits α5 and δ also appear to be downregulated, with persistent reductions reported for both [144,146,149].

**Figure 3:**
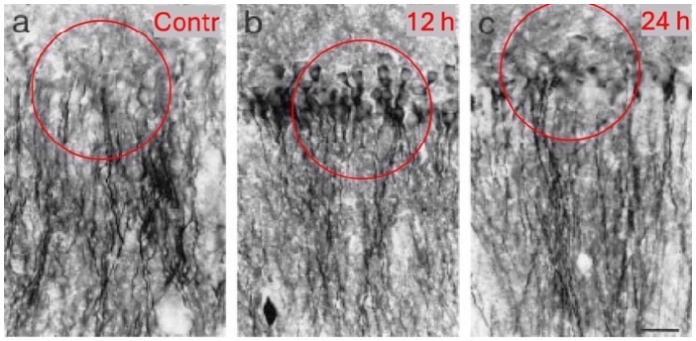
γ2 immunoreactivity in the CA3 pyramidal cell. (**a**) controls, (**b**) 12 h and (**c**) 24 h after KA-induced status epilepticus (SE). Note the significant relocation of the subunit from dendrites to perikarya 12 h after the SE. See also [163,164]. Already 12 h later, as well as after 30 days (not included), the γ2-IR is relocated to the dendrites. The figure was originally published by Schwarzer et al. (1997) in Neuroscience 80, 1001–1017 [146] and part of it was used. The red circles highlight CA3 pyramidal neurons expressing high levels of γ2-IR, 12 and 24 h after KA. Contr, control. Scale bar: 25 µm.

Goodkin et al. (2005 and 2008) and Naylor et al. (2005) [163,164,165] reported internalization of β2/β3 and γ2 from the membrane into the cytoplasm during acute SE. This internalization was accompanied by reduced miniature inhibitory postsynaptic potentials (IPSPs) and was proposed as a mechanism for benzodiazepine resistance. At the same time, tonic currents increased [164]. Notably, γ2-IR does not appear to be degraded; it remains detectable in pyramidal cell dendrites, and γ2 mRNA levels are not increased, arguing against compensatory de novo synthesis. Instead, γ2 seems to be transported from dendrites to the cell bodies during acute SE and are subsequently relocated to the dendrites in the chronic epileptic state. Accordingly, at 10 and 30 days after KA, we observed high γ2-IR in pyramidal dendrites, while perikarya still contained γ2-IR aggregates (Figure 3) [146].

#### 7.4.3. Changes in Interneurons

Interneurons often exhibited strong immunolabeling. Basket cells at the granule layer and pyramidal stratum moleculare were strongly positive for α1, α4, β2, and γ2 and survived up to 30 days after KA. Strong α1-IR was also detected in presumed parvalbumin-positive basket cells in CA1–CA3, although their numbers appeared reduced, as also seen in mouse models [151].

#### 7.4.4. Functional Changes in the Dentate Gyrus

*Acute status epilepticus.* Patch-clamp recordings demonstrated impaired GABAergic function in granule and CA1 pyramidal cells during Li-pilocarpine-induced SE [147,166]. These neurons exhibited reduced sensitivity to benzodiazepines and Zn^2+^. In vitro SE models similarly revealed profound internalization of β2/β3- and γ2-containing receptors, consistent with loss of phasic inhibition [165]. δ expression, however, remained unchanged.

*Chronic epileptic state.* Around 7 to 30 days after the acute SE, recurrent seizures emerged. By this, expression of most subunits has recovered or were upregulated in granule and pyramidal cells (Figure 2 and Figure 3), except that of δ and α5. Accordingly, electrophysiological recordings revealed enhanced maximal GABA responses and strong augmentation by clonazepam in granule cells [133]. In contrast, CA1 pyramidal cells exhibited reduced clonazepam efficacy, consistent with persistent circuitry disruption and the loss of GABA_A_ receptors.

#### 7.4.5. Loss of δ and α5 Subunits Indicates Reduced Tonic Inhibition

A consistent and striking finding across models is the loss of subunit δ in granule cells and of α5 in pyramidal neurons [141,144,145,146,149] at all intervals of kindling and in SE models. Both subunits are key components of extrasynaptic receptors mediating tonic inhibition [162]. GABA can act on synaptic lower-affinity receptors mediating phasic inhibition, or on high-affinity extrasynaptic receptors mediating tonic inhibition. In the dentate gyrus, tonic inhibition typically involves α4δ-containing receptors, while γ2-containing receptors mediate phasic inhibition. In pyramidal cells, tonic inhibition is mediated by α5γ2-containing receptors [162,167]. Reduced δ-subunit expression has also been reported in the dentate gyrus of pilocarpine-treated mice, accompanied by impaired responses to δ-targeting neurosteroids, again arguing for diminished tonic inhibition [148,168]. Immunogold labeling showed γ2-subunits relocating to extrasynaptic sites, forming alternative receptor populations with α1-, α2-, α4-, and β-subunits. Nonetheless, receptor losses at both peri- and extrasynaptic sites were observed, consistent with increased seizure susceptibility [148,169].

In contrast, Naylor et al. (2005) reported acute internalization of β2/β3- and γ2-containing receptors during pilocarpine-induced SE [164]. These subunits are part of GABA_A_ receptors mediating phasic inhibition (e.g., α1β3γ2). Their loss is consistent with impaired phasic inhibition and transient benzodiazepine resistance seen also in patients suffering from a SE [163,164,165,170]. Loss of α5-containing receptors, which mediate tonic inhibition in pyramidal cells [162,171], may also contribute to the heightened seizure susceptibility in these models.

### 7.5. Changes After Traumatic Brain Injury (TBI)

Experimental traumatic brain injury is also considered as an epilepsy model. In our respective study, all GABA_A_ receptor subunit mRNAs (except α4) initially decreased (6 to 24 h after the injury) in all hippocampal areas, both ipsi- and contralaterally to the injury site, but fully recovered after 4 months [172].

## 8. Chloride Channels: Their Role in GABA_A_ Receptor Function and in Epileptogenesis

Under pathological conditions, the inhibitory efficacy of GABA_A_ receptor signaling may be compromised. The hyperpolarizing effect typically associated with GABA_A_ receptor activation is dependent on a chloride gradient, characterized by higher extracellular than intracellular Cl^−^ concentrations. This gradient is established and maintained by the Na-K-2Cl co-transporter isoform 1 (NKCC1) and the K-Cl co-transporter isoform 2 (KCC2). While KCC2 facilitates Cl^−^ extrusion, NKCC1 promotes its influx into the cell [173].

In immature neurons, NKCC1 expression predominates, whereas KCC2 expression is relatively low. This results in elevated intracellular Cl^−^ levels, leading to a net efflux of Cl^−^ upon GABA_A_ receptor activation, thereby inducing membrane depolarization [174]. In contrast, mature neurons exhibit high KCC2 and low NKCC1 expression, promoting a net Cl^−^ influx and a hyperpolarizing response to GABAergic stimulation. The excitatory nature of GABAergic signaling in the developing brain may explain the transient nature of many neonatal epileptic syndromes. In the adult brain, however, pathological events such as deafferentation or trauma can lead to down-regulation of KCC2, impairing Cl^−^ extrusion and causing a depolarizing shift in the GABA_A_ reversal potential [175,176].

In subicular slices obtained from TLE patients, Huberfeld et al. identified a subpopulation of KCC2-deficient neurons exhibiting GABA-induced depolarization during spontaneous interictal discharges [177]. This depolarization was attributed to NKCC1-mediated Cl^−^ accumulation. Notably, pharmacological inhibition of NKCC1 with bumetanide restored the hyperpolarizing effect of GABA and attenuated interictal activity [177]. On the other hand, Karlócai et al. observed a general increased expression of KCC2 containing pyramidal cells and interneurons in the hippocampus of TLE patients as well as in Li-pilocarpine treated mice [178].

## 9. GABA_B_ Receptors in Animal Epilepsy Models

The second major class of GABA receptors consists of GABA_B_ receptors. Unlike GABA_A_ receptors, they are G-protein-coupled receptors that inhibit adenylyl cyclase and voltage-gated Ca^2+^ channels while activating inwardly rectifying K^+^ channels. Together, these actions reduce neuronal excitability through inhibition of dendritic Ca^2+^ signaling, increased postsynaptic membrane conductance, and decreased presynaptic neurotransmitter release [84].

GABA_B_ receptors are heterodimers composed of two principal subunits: GABA_B1_, which contains the agonist-binding domain, and GABA_B2_, which couples to G proteins. Two GABA_B1_ isoforms exist—GABA_B1a_, predominantly presynaptic, and GABA_B1b_, primarily postsynaptic. In addition to forming heterodimers, GABA_B_ receptors can assemble into higher-order multimeric complexes and associate with auxiliary subunits that modulate agonist potency and receptor kinetics. Besides on all principal neurons GABA_B_ receptors are widely expressed by all subtypes of interneurons as shown by co-expression with different neuropeptides and calcium binding proteins [179].

Pharmacologically, GABA_B_ receptor activation exerts anticonvulsant effects in limbic (hippocampal) seizures but can be proconvulsant in thalamic “audiogenic” seizures. The GABA_B_ receptor agonist baclofen blocks kindling [180], whereas GABA_B_ antagonists accelerate it [181]. Mice lacking GABA_B1_ develop spontaneous seizures and epilepsy [182,183]. Downregulation of GABA_B_ receptors enhances inhibitory feedback in the dentate gyrus following KA-induced epilepsy [184].

### GABA_B_ Receptors in KA-Induced Epilepsy

Expression of GABA_B1a_, GABA_B1b_, and GABA_B2_ has been examined at various time points (2–24 h and 30 days) after KA injection using in situ hybridization, immunohistochemistry, and receptor autoradiography with the antagonist [^3^H]-CGP54626A [185]. In this paradigm, early time points (2–12 h) capture responses to acute seizures, whereas later intervals (24 h, 30 d) reflect recurrent seizure activity and epilepsy.

Across hippocampal regions, subunit expression changed in parallel but with region-specific patterns. In the granule cell layer, mRNAs for all subunits declined by ~25% at 2–9 h and then slightly exceeded control levels at later time points. In CA1, GABA_B1_ (the ligand-binding subunit) was expressed at lower levels than GABA_B2_ (the signaling subunit). All subunits remained markedly reduced (≤50% of control) from 2 to 30 days post-KA. In CA3, mRNA levels initially dropped by 50–70% but recovered to ~80% of control. Persistent decreases in CA1 likely reflect neurodegeneration, whereas the partial recovery in CA3 may represent seizure-induced plasticity.

Immunohistochemistry for GABA_B1a/1b_ and GABA_B2_ corroborated these findings: reduced staining in CA1 at 24 h likely resulted from cell loss, whereas increased GABA_B2_ labeling appeared in the stratum lacunosum-moleculare and stratum radiatum of CA3 at 30 days. Notably, strong upregulation of both GABA_B1_ and GABA_B2_ was observed in interneurons—particularly in the dentate hilus—suggesting strengthened GABA_B_-mediated inhibition of interneurons.

A related study using sustained electrical stimulation and kindling found that a single kindling session caused modest increases (4–24%) in hippocampal mRNAs for all subunits [143]. After 7 days, overall GABA_B_ mRNA levels remained elevated (+35%), whereas isoform-specific changes in GABA_B1a/b_ were not significant. Sustained electrical stimulation produced a robust increase in GABA_B2_ mRNA (117–133%) in the granule cell layer, while GABA_B1a/b_ remained unchanged. In CA3, an early decrease (24 h) was followed by recovery at 7 days, and in CA1, all subunits increased despite substantial neuronal loss.

Overall, changes in GABA_B_ receptor mRNA are modest but may have important functional consequences at the single-cell level. GABA_B2_ expression appears more sustained and may be key to restoring inhibitory balance. Decreases in CA1 likely reflect neurodegeneration or impaired receptor function, while later increases suggest compensatory upregulation in surviving neurons.

Haas et al. (1996) demonstrated that GABA receptors regulating GABA release are downregulated after KA-induced seizures, enhancing synaptic inhibition [184]. These electrophysiological findings are consistent with neurochemical studies showing reduced GABA_B1_ and GABA _B2_ [143] mRNA levels and decreased [^3^H]-CGP54626 binding in granule and pyramidal cells of KA-treated rats [185].

## 10. Changes in GABA Receptors in Human TLE

Studies of GABA_A_ receptor subunit expression are typically performed on hippocampal specimens from patients who have undergone unilateral hippocampal resection for therapeutic reasons. Control tissue is generally collected at routine autopsy from individuals who died of causes unrelated to epilepsy or ischemia. Interpretation of histochemical studies is often complicated by the pronounced neurodegeneration found in many TLE patients, including substantial neuronal loss in CA1 and CA3 (Ammon’s horn sclerosis). Granule cells and CA2 pyramidal neurons are relatively preserved, although granule cell loss can reach 50% [159,186,187]. With progressive granule cell loss, the granule cell layer may disperse and occasionally form a double layer [159,188]. The subiculum appears relatively resistant. Distinguishing whether reductions in receptor binding reflect cell loss alone or also receptor downregulation can be challenging. Moreover, tissue shrinkage secondary to cell loss can sometimes artifactually increase apparent binding density.

In contrast, some TLE cases exhibit minimal hippocampal neurodegeneration despite severe epilepsy (non-lesional or “non-sclerotic” TLE). Many of these patients also benefit from epilepsy surgery.

### 10.1. GABA_A_ Receptor Binding in TLE Patients

Koepp et al. compared ex vivo [^3^H]flumazenil autoradiography with [^11^C]flumazenil PET in the same TLE patients [131,132,189]. Both methods yielded similar findings: reduced [^11^C]flumazenil binding corresponded closely to the extent of Ammon’s horn sclerosis.

In the dentate gyrus—particularly its molecular layer—and in the subiculum, both regions with comparatively little neurodegeneration, GABA_A_ receptor binding appears unchanged or even increased, as shown by autoradiography [190,191]. Interestingly, one PET study reported reduced [^11^C]flumazenil binding in the insular cortex [192], possibly related to neuropsychological alterations in TLE patients.

### 10.2. Changes in GABA_A_ Receptor Subunits in TLE Patients

*α-subunits.* Loup et al. (2000) observed modest increases in α1 and α2 expression in the subgranular region of the dentate molecular layer in TLE specimens, with α2 expression also moderately elevated in other molecular-layer regions [152]. In our studies, α1-IR was decreased in the molecular layer of non-sclerotic patients and even more markedly reduced in patients with hippocampal sclerosis [153]. We did not assess α2. By contrast, α3 expression was dramatically increased in the dentate molecular layer, subiculum, and presubiculum [153], indicating a shift from α1/α2-containing receptors toward α3-containing receptors.

**Figure 4:**
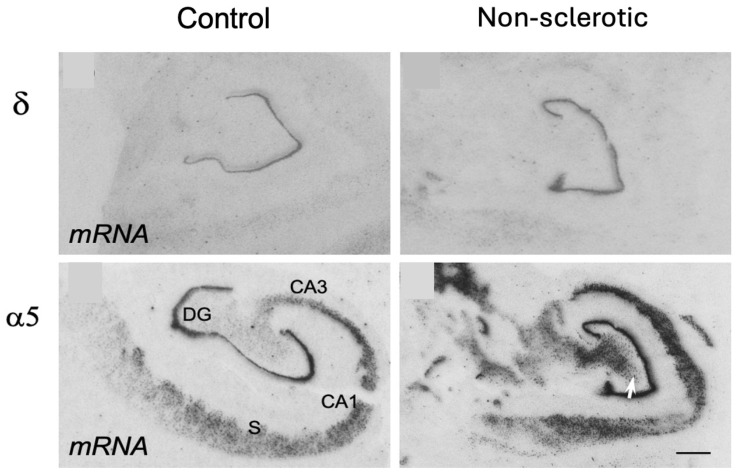
Expression of subunits α5 and δ in the hippocampus of non-sclerotic samples from TLE patients. These subunits are contained in GABA_A_ receptors that are extrasynaptically located and mediate tonic inhibition. In contrast to human TLE, these subunits are downregulated in rodent models of TLE. The Figure is part of Figure 1, originally published by Sperk et al., in Brain Communications (2021) [154]. Note the increased δ mRNA in granule cells of the non-sclerotic TLE sample, and α5 mRNA in granule cells, CA3 to CA1 pyramidal cells up to the subiculum and possibly also in hilar interneurons (white arrow) that are strongly enhanced in the TLE specimen. DG, dentate gyrus (granule cell layer); S, subiculum. Scale bar: 200 µm.

Using in situ hybridization and immunohistochemistry, we also detected marked increases in α4 and α5 subunit mRNAs in the granule cells/molecular layer, subiculum, and parasubiculum of both sclerotic and non-sclerotic patients. In non-sclerotic specimen, α5, but also α4, were strongly expressed throughout the pyramidal cell layer—especially in CA2 of non-sclerotic patients, but also in tissue with Ammon’s horn sclerosis [154]. Binding of the α5-selective ligand [^3^H]L-655.708 was similarly increased in the dentate molecular layer [154] (Figure 4). These findings agree with a PET study using [^11^C]Ro15-4513, which showed increased α5-associated signal in TLE patients and decreased α1, α2, and α3 expression, suggesting a shift toward α5-containing receptors [193]. This contrasts sharply with animal epilepsy models, which consistently report reduced α5 expression in pyramidal cells of epileptic rats [145,146,149].

*β-subunits.* GABA_A_ receptor β-subunits, together with α-subunits, form the GABA binding site. IR for all three β-subunits was increased in the molecular layer of both sclerotic and non-sclerotic specimens, with β3 showing the most pronounced upregulation in sclerotic tissue [153]. Whereas β3-IR was distributed throughout the molecular layer, β1- and β2-IR were more concentrated in the inner molecular layer, corresponding to the location of parvalbumin-positive basket cell terminals. This pattern may reflect a compensatory response to the partial degeneration of these interneurons. Strong expression of all β-subunits was also observed in the subiculum and parasubiculum, which are major hippocampal output regions. Overall, in regions with limited neurodegeneration, GABA_A_ receptor subunits appear to be either unchanged or markedly upregulated.

*γ2- and δ-subunits.* γ2-IR was reduced in the dentate molecular layer of both sclerotic and non-sclerotic tissue compared with controls [153]. In non-sclerotic samples, γ2-IR was concentrated in the supragranular molecular layer. In the Ammon’s horn, γ2-IR was markedly decreased, likely due to neuronal loss. Loup et al. also reported minimal changes in the dentate gyrus aside from a modest increase in γ2 within the granule cell layer of sclerotic specimens [152]. In striking contrast to animal models [144,145,148], δ-subunit expression—together with α4—was significantly elevated in granule cells, the molecular layer, and the subiculum of TLE patients with Ammon’s horn sclerosis [154].

### 10.3. GABA_B_ Receptors in Human TLE

Changes in GABA_B_ receptors were assessed using the GABA_B_-selective ligand [^3^H]CGP62349 and by examining GABA_B1_ and GABA_B2_ expression via in situ hybridization and immunohistochemistry in surgically obtained TLE tissue—with and without Ammon’s horn sclerosis—and in autopsy controls [194,195].

**Figure 5:**
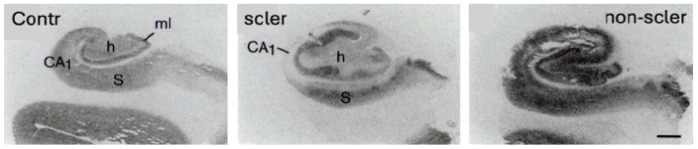
Receptor autoradiography with the GABA_B_ receptor antagonist [^3^H]-CGP546266 in specimen from controls, and TLE patients with (scler) and without (non-scler) Ammon’s horn sclerosis. The receptors are significantly upregulated, as also shown by in situ hybridization [194]. The Figure was originally published by Furtinger et al. (2003) [194]. CA_1_ subfield of the cornu ammonis; h, dentate hilus; S, subiculum; ml, molecular layer of the dentate gyrus. Scale bar: 2 mm.

In patients with Ammon’s horn sclerosis, [^3^H]CGP62349 binding was reduced in CA1–CA3 pyramidal neurons, most likely due to neuronal loss. The subiculum and dentate molecular layer were relatively preserved and showed strong labeling. In non-sclerotic patients, dramatic increases in labeling were detected in the dentate hilus and throughout the pyramidal cell layer (Figure 5). After correcting for cell loss, these increases remained significant in non-sclerotic samples and in the hilus and molecular layer of the dentate gyrus [194,195].

In situ hybridization results were similar. In non-sclerotic specimens, substantial increases in GABA_B1_ and GABA_B2_ mRNA were observed in the subiculum, CA3, granule cells, and the dentate molecular layer. After adjusting for cell loss, GABA_B1_ and GABA_B2_ mRNA increased by 173% and 344%, respectively, in sclerotic tissue, and by 107% and 189% in non-sclerotic tissue. Binding data showed also statistically significant increases after the same corrections [194,195]. The strong hybridization and binding signals in the dentate hilus indicate that GABA_B_ receptors are also upregulated in interneurons, consistent with findings in rat tissue [185].

As in the KA model, in human TLE GABA_B_ receptor binding was concentrated in dendritic regions of principal neurons. These receptors may be predominantly postsynaptic and contribute to inhibition of pyramidal neurons, serving as an endogenous anticonvulsant mechanism. Because pyramidal neurons receive extensive excitatory input from other pyramidal neurons within the tri-synaptic circuit, presynaptic GABA_B_ receptors would additionally inhibit glutamate release and thereby counteract excitatory transmission.

## 11. Summary of Epilepsy-Induced Changes in GABA Receptors and Differences Between Animal Models and Human TLE

Kindling models reproduce recurrent seizures through repeated electrical stimulation, but they do not produce spontaneous seizures and show no evidence of neurodegeneration. In contrast, TLE models more closely mirror the condition of TLE in humans, exhibiting pronounced neurodegeneration, including substantial loss of pyramidal cells in CA1 and CA3 and loss of interneurons. Granule cells are relatively preserved, although up to 50% may be lost. Notably, pyramidal cells in CA2, as well as cells in the subiculum and parasubiculum, are largely spared. This pattern is also observed in animal TLE models, which notably do not show granule cell loss.

After kindling for 7 or 30 days, when the seizure threshold is reduced, mRNAs encoding subunits α4, β2, β3, and γ2 are upregulated in the granule cell layer, whereas α2, α3 and α5 remain largely unchanged and δ-subunit mRNA is significantly reduced [142,145]. In CA1, α2, β1, and γ2 mRNAs are increased.

Findings in animal models that induce chronic epilepsy are similar. In KA-induced epilepsy model (30 days post-injection), α1, α3, and α4 mRNAs are increased in the dentate molecular layer, whereas α2 remains unchanged and α5 is decreases. The β2 and β3 subunits are upregulated (Figure 2, Table 1), γ2 remains unchanged and δ is reduced across all post-KA time points. In CA1 and CA3 pyramidal layers, all α-, β- and γ2-subunit mRNAs decrease (except for α1 in CA3), consistent with pyramidal cell loss and neurodegeneration [144].

In the Li-pilocarpine mouse model, Peng et al. reported increased α4, a non-significant increase in γ2, and a robust decrease in δ in the granule cell layer [148]. These findings align with Fritschy et al. (1999), who observed increases in α3, α5, β2/β3 and γ2 expression with unchanged α1 and α2 and partially with Brooks-Kayal et al. (1998), who reported increased α3, α4, β3 and δ mRNAs [147,150]. Similarly, Bouilleret et al. described increased IR for α1, α3, α5, and γ2 in the dentate molecular layer following unilateral intrahippocampal KA injection, suggesting a shift from α2 to other α-subunits [151]. Collectively, increased expression of α3, α4, β3 and likely of also α1 and γ2 indicates enhanced GABA_A_ receptor expression consistent with receptor-binding data from kindled rats [138]. In contrast, there is clear evidence in rodent models for selective loss of α5 subunits in pyramidal cells (beyond that attributable to neurodegeneration) and of δ-subunits in granule cells.

In TLE patients, changes in GABA_A_ receptor subunits in the dentate molecular layer closely mirror those in rodent models [152,153,154]. Significant increases in α2, α3, α4, α5, β1, β2, β3, γ2, and δ- but not α1- have been reported, indicating generalized GABA_A_ receptor upregulation and a shift away from α1-containing receptors, particularly toward α3. Notably, many interneurons were well labeled for α1 and β2 [150,153].

In contrast, in CA1 of TLE patients with Ammon’s horn sclerosis show marked reductions in α1, α3, β1, β3, and γ2 [152]. In non-sclerotic CA1 tissue, α3-IR was strongly increased in the pyramidal cell layer. Similarly, the subiculum and parasubiculum—especially in non-sclerotic tissue—exhibit overexpression of α3-, α4-, α5, β2-, β3-, γ2-, and δ- subunits [152,153,154].

Electrophysiological studies of cortical cells from TLE patients demonstrate clonazepam efficacy comparable to that in adult rats [133]. In epileptic rats, enhanced GABAergic transmission and altered allosteric modulation of GABA_A_ receptors by benzodiazepines, barbiturates and by zinc ions have been observed in the dentate gyrus [133,147,166]. During acute lithium/pilocarpine-induced SE, dentate granule cells show reduced sensitivity of GABA-evoked currents to benzodiazepines and zinc, but not to barbiturates [135,147].

During chronic epilepsy with spontaneous seizures, several weeks after the initial insult, clonazepam exhibited enhanced effects on GABA currents. In contrast, zolpidem—which selectively targets α1-containing GABA_A_ receptors—showed reduced efficacy in both acute and chronic phases. These observations suggest a progressive shift in receptor composition from α1- to α2-, α3- or α4- subunits. Supporting this, histochemical studies revealed early decreases in α2, α3, β1, β3 and γ2 mRNA expression (6 h after KA) and reductions in α2- and γ2-IR during the *SE* (12 h after KA), followed by a general upregulation in chronic epilepsy (30 days after KA). Interestingly, in epileptic rats, increased zinc-mediated inhibition—possibly due to release from sprouted mossy fibers—may paradoxically disrupt GABAergic signaling by over-activating GABA_A_ receptors in the dentate molecular layer [135].

A notable divergence between animal models and human TLE concerns the subunits mediating tonic inhibition. In rodent models, δ-subunits in granule cells and α5-subunits in pyramidal cells are decreased, whereas in human TLE they are increased, indicating enhanced tonic inhibition [154]. Tonic inhibition is mediated by extrasynaptic GABA_A_ receptors—α4β3δ in the dentate molecular layer and α5β3γ2 in pyramidal neurons—and provides broad inhibitory control across neuronal and glial compartments [196].

The pronounced upregulation of α5-containing receptors in pyramidal cells of TLE patients highlights the potential therapeutic avenue. Pharmacological agents, including benzodiazepine- or β-carboline-related compounds that selectively target α5-receptors, could be explored [197]. Several non-convulsive inverse agonists exist and may serve as leads for developing full agonists [198,199].

The findings on GABA_B_-receptor expression are consistent between rodent models and human TLE. While GABA_B_-receptor subunits are initially downregulated during acute *SE*, they subsequently recover—or even become overexpressed—once animals develop spontaneous recurrent seizures (epilepsy). In human TLE, GABA_B_-receptor subunits are markedly overexpressed throughout the hippocampus, particularly in non-sclerotic samples, as shown by mRNA analyses and receptor-binding studies. These findings suggest that GABA_B_ receptors contribute to enhanced GABAergic transmission, likely via postsynaptic receptors on pyramidal cell dendrites.

## 12. Conclusions

This review highlights the diverse mechanisms by which the GABAergic system protects the brain against seizure progression, focusing on neuropathological and neuropharmacological evidence from animal models and human TLE, while largely excluding clinical and single-cell electrophysiological studies.

Key findings include increased production of the GABA-synthesizing enzymes GAD65 and GAD67, their ectopic expression in glutamatergic mossy fibers, and multiple adaptive changes in GABA_A_ and GABA_B_ receptors. Notably, GABA_B_ receptors are upregulated in the epileptic human brain and expression of GABA_A_ and receptor subunits mediating tonic inhibition is enhanced.

Crucially, substantial differences in receptor subunit expression exist between animal models of TLE and the human condition. These differences highlight the importance of developing therapeutic strategies that selectively target specific GABA receptor subtypes.

## Figures and Tables

**Table 1:** Changes in GABA_A_ receptor subunits in animal models and in human TLE. The data represent changes in in situ hybridization signals in the granule cell layer of the dentate gyrus; changes in IR refer to the dentate molecular layer. Symbols are used for the following ranges of in situ-hybridization signals (% of control): ++, >150; +, 115–150; (+), 105–114 (statistically insignificant), ±, 96–104 (varying); =, (clearly unchanged); (-), 91–95 (statistically insignificant); -, 50–90; - -, <50 (both significant changes). Changes in immunoreactivity (IR) were estimated accordingly in the dentate molecular layer. * strongly reduced in pyramidal cell layer.

			Animal Models of TLE																TLE in Humans			
		**KA (rat)** Dorsal [144] Ventral [145]			**KA (rat)** [146]			**Li/pilo (rat)** [147]			**Li/pilo** **(mouse)** [148,149,150]			**KA** **(mouse)** Intrahip [151]		**Kindling** **(rat)** [140,141]		**Electrical** **stim** **(rat)** Ventral [143]		Loup et al. [152]	Pirker et al. [153]	Sperk et al. [154]
	
		*Time after status epilepticus*														*Time of terminating kindling*			*Human TLE*			
		**12 h**	**7–30 d**		**24 h**	**30 d**		**24 h**	**>30 d**		**24 h**	**30 d**		**>30 d**		**24 h**	**28 d**	**7 d**		IR	IR mRNA	IR, mRNA, Receptor binding
		mRNA			IR			mRNA						IR		mRNA		mRNA				
α**1**		++	+		(-)	++		- -	-			++		++		=	=	=		+	- -	
α**2**		- -	=		-	++			=			-		-		+	+	+		+		
α**3**		- -	+		-	=		++	+			+		+		=	=			=	++	
α**4**		+	(+)		=	++		+	++		-	++				+	=	(+)				++
α**5**		- -	- -		-	± *						- -		(+)		=	(-)	=				++
																						
β**1**		+	+		-	±		- -	-							=	+	(+)			++	
β**2**		±	++		+	++										=	+	(+)		+	++	
β**3**		-	±		=	++		++	++							+	+	(+)		+	++	
																						
γ**2**		-	=		(+)	++					-	+		++		+	+	(+)			±	
**δ**		- -	- -		- -	- -		++	++		- -	- -					=	- -				++

## Data Availability

No new data were created or analyzed in this study. Data sharing is not applicable to this article.

## Abbreviations

The following abbreviations are used in the manuscript:

BBB	blood–brain barrier
CCK-8	cholecystokinin octapeptide
EEG	electro encephalogram
GABA	γ-aminobutyric acid
GABA_A_	GABA_A_ receptor
GABA_B_	GABA_B_ receptor
GIRK	G-protein-activated inwardly rectifying potassium channels
ILAE	International League against Epilepsy
GAD	glutamate decarboxylase
GAD65, GAD67	isoforms of glutamate decarboxylase
GAD25	embryonic glutamate decarboxylase
IR	immunoreactivity
KA	kainic acid
NKB	neurokinin B
KCC2	K-Cl co-transporter isoform 2
NKCC1	Na-K-2Cl co-transporter isoform 1
NPY	neuropeptide Y
O-LM cells	**o**riens to the stratum **l**acunosum **m**oleculare cells
SE	status epilepticus
TLE	temporal lobe epilepsy
VIP	vasoactive intestinal peptide
VGAT	vesicular GABA transporter
